# Tailoring Triple Filler Systems for Improved Magneto-Mechanical Performance in Silicone Rubber Composites

**DOI:** 10.3390/polym15102287

**Published:** 2023-05-12

**Authors:** Vineet Kumar, Md Najib Alam, Manesh A. Yewale, Sang-Shin Park

**Affiliations:** School of Mechanical Engineering, Yeungnam University, 280, Daehak-ro, Gyeongsan 38541, Republic of Korea; vineetfri@gmail.com (V.K.); mdnajib.alam3@gmail.com (M.N.A.); maneshphd@gmail.com (M.A.Y.)

**Keywords:** multi-wall carbon nanotube, silicone rubber, stretchability, energy harvesting, magnetic sensitivity

## Abstract

The demand for multi-functional elastomers is increasing, as they offer a range of desirable properties such as reinforcement, mechanical stretchability, magnetic sensitivity, strain sensing, and energy harvesting capabilities. The excellent durability of these composites is the key factor behind their promising multi-functionality. In this study, various composites based on multi-wall carbon nanotubes (MWCNT), clay minerals (MT-Clay), electrolyte iron particles (EIP), and their hybrids were used to fabricate these devices using silicone rubber as the elastomeric matrix. The mechanical performance of these composites was evaluated, with their compressive moduli, which was found to be 1.73 MPa for the control sample, 3.9 MPa for MWCNT composites at 3 per hundred parts of rubber (phr), 2.2 MPa for MT-Clay composites (8 phr), 3.2 MPa for EIP composites (80 phr), and 4.1 MPa for hybrid composites (80 phr). After evaluating the mechanical performance, the composites were assessed for industrial use based on their improved properties. The deviation from their experimental performance was studied using various theoretical models such as the Guth–Gold Smallwood model and the Halpin–Tsai model. Finally, a piezo-electric energy harvesting device was fabricated using the aforementioned composites, and their output voltages were measured. The MWCNT composites showed the highest output voltage of approximately 2 milli-volt (mV), indicating their potential for this application. Lastly, magnetic sensitivity and stress relaxation tests were performed on the hybrid and EIP composites, with the hybrid composite demonstrating better magnetic sensitivity and stress relaxation. Overall, this study provides guidance on achieving promising mechanical properties in such materials and their suitability for various applications, such as energy harvesting and magnetic sensitivity.

## 1. Introduction

In magneto-rheological elastomers (MREs), an important constituent is elastomers. There are different types of elastomers used, such as natural rubber (NR) [1], styrene-butadiene rubber (SBR) [2], nitrile butadiene rubber (NBR) [3], and silicone rubber (SR) [4]. Among them, SR is frequently used as an elastomer matrix in MREs. Various studies have shown that SR is a fascinating matrix that is well-suited for use in MREs due to its soft nature, low viscosity, ease of curing, and ease of processing [4,5]. There are various possible types of silicone rubbers depending on the type of vulcanization used, with single components, such as room-temperature silicone rubber [6], and two-component silicone rubber or high-temperature vulcanized silicone rubber [7]. Among them, RTV-SR is more promising due to its ease of processing and soft nature, and is thus explored in this work.

The properties of MREs are affected by the types of additives used [8]. Magnetic fillers and reinforcing fillers are commonly used as additives in MREs [9,10]. Magnetic fillers can be classified based on their particle size, shape, or surface area [11]. Among the various types of magnetic fillers used in MREs, carbonyl iron particles (CIP) with different morphologies and sizes are most commonly used [12,13]. Studies have shown that CIP can act as a favorable magnetic filler due to its favorable oval morphology and small particle size [13,14]. Other types of magnetic particles include iron oxides ranging from micron-sized to nano-sized [15]. In addition to magnetic fillers, reinforcing fillers from various classes are also used. The most promising reinforcing fillers reported in the literature over the last two to three decades are nanocarbon black (NCB) [16], carbon nanotubes (CNTs) [17], graphene (GR) [18], and clay minerals [19]. Studies have shown that CNTs are a fascinating reinforcing filler that lead to a drastic increase in mechanical and electrical properties at loadings lower than 5 phr, especially in elastomer matrixes [20]. Several studies have reported the use of CNTs as reinforcing additives in MREs [21,22]. In a few studies, GR was used as a reinforcing agent in MREs [22,23]. However, the reinforcement provided by using clay minerals in MREs is not yet fully understood and is thus explored in the present work. 

The mechanical stiffness of the composites used in MREs depends upon the formation of the microstructure under a magnetic field [24]. The non-magnetic fillers are dispersed randomly while the magnetic filler is oriented in the direction of the magnetic field [25,26]. The orientation of the magnetic fillers depends upon the magnitude of the magnetic field, the time of exposure to the magnetic field, and the type of magnetic filler used in such composites [27]. In addition to these parameters, the mechanical properties also depend upon the type of non-magnetic filler, its morphology, its shape, size, and the aspect ratio of the non-magnetic filler [28]. In some cases, a hybrid filler containing both magnetic and non-magnetic fields was found to be promising [29], and is thus explored in the present work. 

Numerous studies have been conducted on the use of hybrid fillers in MREs [30]. These hybrid fillers can be either both magnetic or a combination of one magnetic and one reinforcing filler [31]. However, the use of triple hybrid fillers, which consist of two reinforcing and one magnetic filler, is not fully understood in MREs, and, therefore, this study aims to explore their properties. Additionally, the stress–strain curves of composites containing these hybrid fillers require further investigation, which is also explored in this study. The present work assesses the synergistic effect of these triple hybrid fillers. It should be noted that MWCNT is a promising reinforcing filler; however, its use in high amounts significantly reduces the stretchability of composites. Therefore, the addition of MT-Clay is proposed to improve this mechanical property without significantly affecting the modulus. Furthermore, EIP was added to make the composites magnetically active. Hence, the use of these three fillers is justified and presented in this work. This study also investigates the magneto-mechanical behavior of individual fillers and their hybrid filler systems.

## 2. Materials and Methods

### 2.1. Materials

The RTV-Silicone rubber used in this work was obtained from Shin-Etsu Chemical Corporation Ltd., Tokyo, Japan. It was purchased under the commercial name “KE-441-KT” and has a transparent appearance. The vulcanizing material used was also obtained from Shin-Etsu Chemical Corporation Ltd., Tokyo, Japan, and its commercial name is “CAT-RM.” The MWCNT used, which has the commercial name CM-100, was purchased from Hanwha Nanotech Corporation Ltd., Seoul, Republic of Korea. The clay minerals used (Montmorillonite K10) have a surface area of 220–270 m^2^/g and were purchased from Sigma Aldrich, St. Louis, MO, USA. The electrolyte iron particles (EIP) used, which have the commercial name “Fe#400,” were purchased from Aometal Corporation Limited, Gomin-si, Republic of Korea. The EIP particles were irregular in shape and had a greyish color with micron-sized particles in the range of 10–12 µm. The elemental composition of the EIP was 98.8% iron with traces of nitrogen, oxygen, and carbon. The mold-releasing agent was purchased from Nabakem, Pyeongtaek-si, Republic of Korea.

### 2.2. Characterizations of Fillers and Composites

The morphology of the nanofillers used in this study was investigated using a SEM microscope (S-4800, Hitachi, Japan). Prior to imaging, the samples were sputtered with platinum for 2 min to make their surface conductive. The dispersion of fillers in the composite samples was evaluated using an optical micrograph (Sometech Inc., Seoul, Republic of Korea). To study filler dispersion in the rubber matrix using SEM, the cylindrical samples used for measuring the compressive mechanical properties were sectioned into approximately 0.2 mm thick slices. These slices were then mounted on an SEM stub and their surfaces were coated with platinum to make them conductive. Finally, SEM measurements were taken. The mechanical properties under compressive and tensile strain were evaluated using a universal testing machine (UTS, Lloyd instruments, West Sussex, UK). The mechanical properties under compressive strain were determined using cylindrical samples at a strain rate of 4 mm per minute from 0 to 35% strain. Similarly, mechanical properties under tensile strain were determined using a UTS machine at a strain rate of 200 mm per minute using a dumbbell-shaped specimen. The thickness of the dumbbell-shaped specimen was 2 mm and the gauge length was 25 mm. These mechanical tests were performed according to DIN 53 504 standards. Piezoelectric tests were performed using a UTS machine under cyclic loads (Lloyd Instruments, West Sussex, UK). The output voltage generated through the specimen was recorded using a digital multi-meter (Agilent 34401A, Santa Rosa, CA, USA). The energy harvesting sample was composed of MWCNT, MT-Clay, EIP, and their hybrid. The magneto-mechanical properties were tested at 30% strain using UTS under compressive strain. The procedure for magnetic sensitivity and stress relaxation under a magnetic field involved am investigation using cylindrical samples (10 mm thickness and 20 mm diameter) under 10% compressive strain. The strain rate was 1 mm/min for 5 s of magnetic switching to complete one cycle. Both magnetic sensitivity and stress relaxation were studied under on–off switching of the magnetic field at 100 mT.

### 2.3. Preparation of Rubber Nanocomposites

**Scheme 1:**
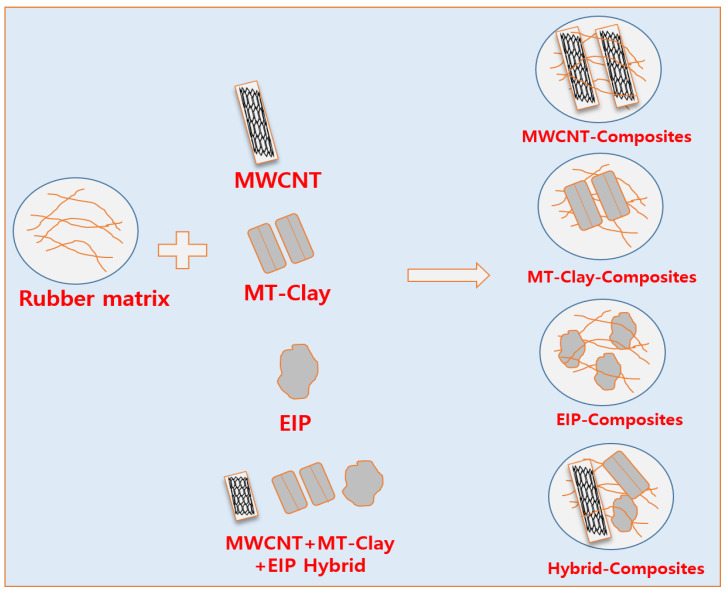
Fabrication of different types of MREs.

The fabrication process of the MREs was initiated by following the optimized procedure from a previous study [32]. First, a predetermined amount of liquid silicone rubber was taken in a beaker, and then a known amount of different grades of nanofillers (Table 1) were added to the liquid rubber. The mixture was then stirred for 10 min. After the nanofiller–rubber mixing phase, 2 phr of the vulcanizing agent was added, and the final rubber composite was poured into molds. The molds were manually pressed and left for 24 h for vulcanization at room temperature (25 °C). Finally, the samples (Scheme 1) were removed from the molds and tested for various properties to assess their suitability for industrial MRE applications.

## 3. Results and Discussion

### 3.1. Morphology of the Filler

**Figure 1:**
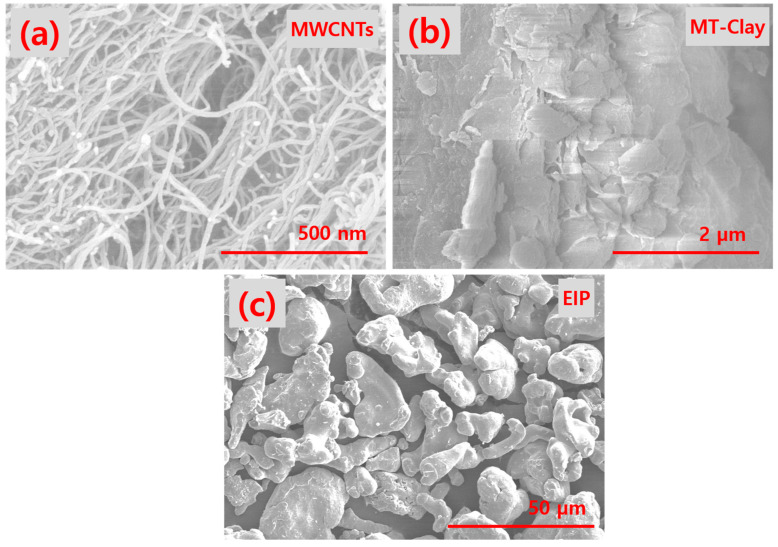
SEM images of (**a**) MWCNT particles; (**b**) MT-Clay particles; (**c**) EIP particles.

It is well known that the morphology of nanofillers greatly affects the properties of composites [33]. Fillers with small particle sizes and favorable shapes have better and more uniform dispersion, leading to a greater impact on the composites [34]. Figure 1 illustrates the morphology of the different nanofillers used as fillers in this study. The morphologies range from one-dimensional (1D) MWCNT to 2D MT-Clay and 3D EIP. MWCNT has a tube-shaped morphology, which allows for easy dispersion and formation of continuous filler–filler contacts with a much lower MWCNT content in a rubber matrix. Furthermore, its high surface area and small particle size provide a large interfacial area, allowing more polymer chains to adsorb to its surface [35]. MT-Clay has a sheet-like morphology, making it easy to disperse in the rubber matrix. It is considered a nanofiller since its particle size is in the nanometer range. Both MWCNT and MT-Clay are ideal fillers and significantly improve composite properties in small amounts in the rubber matrix. Lastly, EIP has an irregular morphology and large particle size, likely in the micrometer range. Due to its large particle size, it has relatively poor reinforcing abilities in lower amounts and is thus used in higher amounts to achieve optimal reinforcement in a composite.

### 3.2. Filler Dispersion in SR Matrix Using Optical and SEM Micrographs

**Figure 2:**
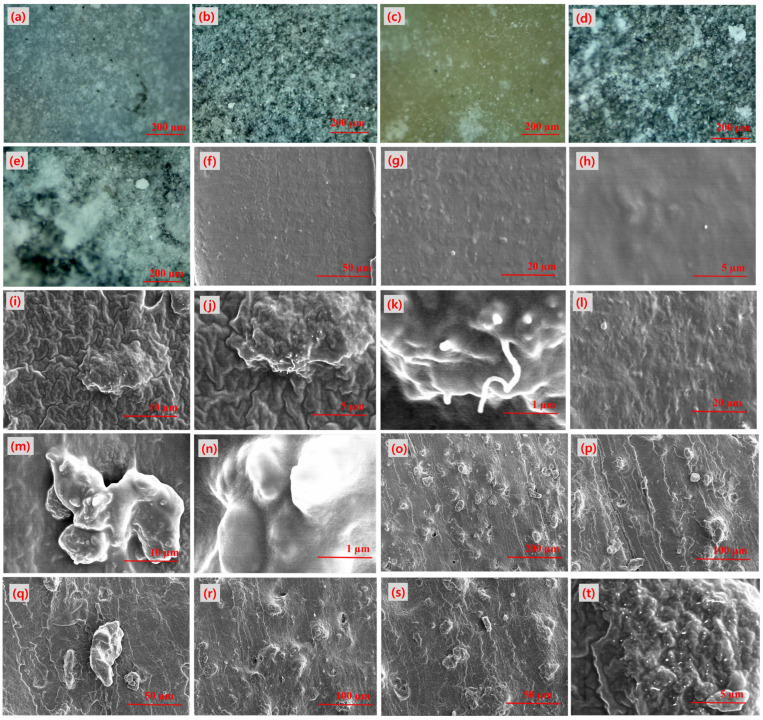
Optical micrographs of different specimens: (**a**) control sample; (**b**) 3 phr MWCNT; (**c**) 6 phr MT-Clay, (**d**) 60 phr EIP, (**e**) 60 phr Hybrid. SEM micrographs at different resolutions: (**f**–**h**) control sample; (**i**–**k**) MWCNT-filled composites; (**l**–**n**) MT-Clay-filled composites; (**o**–**q**) EIP-filled composites; (**r**–**t**) Hybrid-filled composites.

The dispersion of filler in composites is known to affect their properties [36]. A uniform filler dispersion leads to improved overall properties, while composites with poorly dispersed filler have poorer properties [37]. Therefore, this study investigates filler dispersion using optical microscopy and reports its correlation with mechanical properties. The presented optical micrographs in Figure 2 show good filler dispersion for all fillers except for MT-Clay and the hybrid filler. Figure 2a displays the micrographs of the control sample without any filler [38], indicating the absence of filler. Figure 2b shows the uniform dispersion of the MWCNT filler in the rubber matrix, and filler-rich zones with no aggregation are observed, justifying the promotion of MWCNT-based composites as having better properties. Similarly, Figure 2d shows the optical micrographs of the EIP-filled rubber matrix, showing the uniform dispersion of EIP particles and their correlation with improved mechanical properties such as modulus. As reported earlier, Figure 2c,d shows the optical micrographs of the MT-Clay and hybrid composites, respectively. The images also show improved filler dispersion, as in other filled composites, but few filler aggregates or filler-rich zones are reported [39]. The optical micrographs alone do not provide convincing evidence for studying filler dispersion, particularly due to the lack of high-resolution information about the fillers. As a result, filler dispersion was further analyzed using SEM microscopy at both lower and higher resolutions. Figure 2f–h displays SEM images of the control sample at different resolutions, where the absence of filler particles with a smooth surface can be seen. Figure 2i–k shows SEM images of MWCNT-filled composites, where the low-resolution images indicate an increase in the roughness of the rubber matrix. Moreover, at a higher resolution, the CNTs can be seen protruding out from the rubber matrix. Figure 2l–n displays SEM micrographs for MT-Clay-filled composites, where both lower and higher-resolution images show the presence of filler aggregates, supporting the conclusion that these composites have poorer mechanical properties. Next, Figure 2o–q exhibits the dispersion of EIP particles in rubber composites, where micron-sized EIP particles were uniformly dispersed. Furthermore, the high-resolution image shows good adhesion between the EIP and rubber particles. Finally, the study of hybrid fillers is presented in Figure 2r–t, where the different filler particles are uniformly dispersed in the composite, resulting in better properties in the hybrid composites.

### 3.3. Mechanical Properties of Rubber Nanocomposites under Compressive Strain

**Figure 3:**
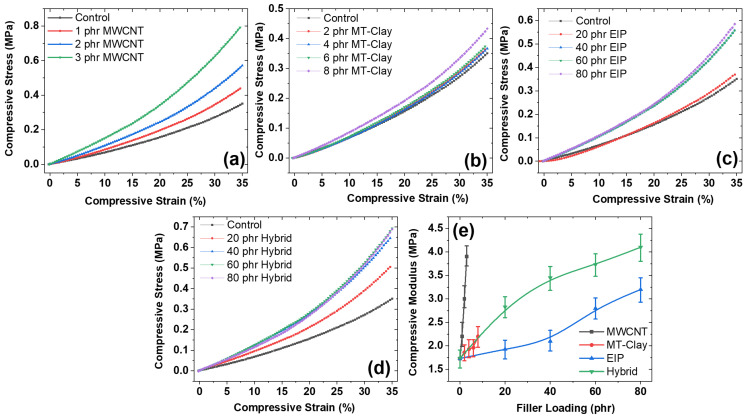
Mechanical properties under compressive strain: (**a**) stress–strain of MWCNT; (**b**) stress–strain of MT-Clay, (**c**) stress–strain of EIP; (**d**) stress–strain of hybrid filler; (**e**) compressive modulus of different composites under different loading conditions.

The mechanical properties of composites depend on various parameters, such as the type of filler, the type of polymer matrix, and the type of applied strain during testing [40,41]. Certain mechanical properties, such as stretchability and stiffness, play an important role in prospective applications, such as flexible electronics [42]. The stress–strain curves under compressive strain from 0–35% are shown in Figure 3a–d. The maximum compressive strain of 35% was chosen due to the fracture of the cylindrical sample after 35% compressive strain. The stress–strain behavior of different composites indicates that the stress increases linearly up to 15% and then increases exponentially. This behavior is attributed to the increase in packing fractions of the filler and rubber particles under higher compressive strain [43]. Additionally, the stress increases in all composites with an increase in filler content, which is attributed to improved filler networking, filler–filler, and rubber–filler interactions [44,45].

In Figure 3e, the impact of filler loading and filler type on compressive modulus is illustrated. Firstly, the effect of different filler types on mechanical properties was examined. It was observed that MWCNT, with its small particle size, high surface area, and high aspect ratio, demonstrated a promising reinforcing effect on the silicone rubber matrix. These MWCNT features, such as (a) the large aspect ratio, which helps to improve filler–filler interconnection at a lower filler loading [46]; (b) small particle size and large surface area, which provide a greater interfacial area for more rubber polymer chains to get adsorbed onto the filler surface [47]; and (c) higher interfacial area, which allows improved stress transfer at the polymer–filler interface [48]. It was also observed that MT-Clay provides a medium level of reinforcement which is higher than EIP and much lower than MWCNT. The poor reinforcement of EIP particles is due to their micron-sized particles and small surface area, which translates to a lower interfacial area. All of these EIP qualities make it an inferior source of reinforcement. Additionally, a higher amount of EIP filler is required to obtain optimum reinforcement, which is an order of magnitude higher (40 phr) than that of MWCNT (1 phr).

### 3.4. Mechanical Properties under Tensile Strain

**Figure 4:**
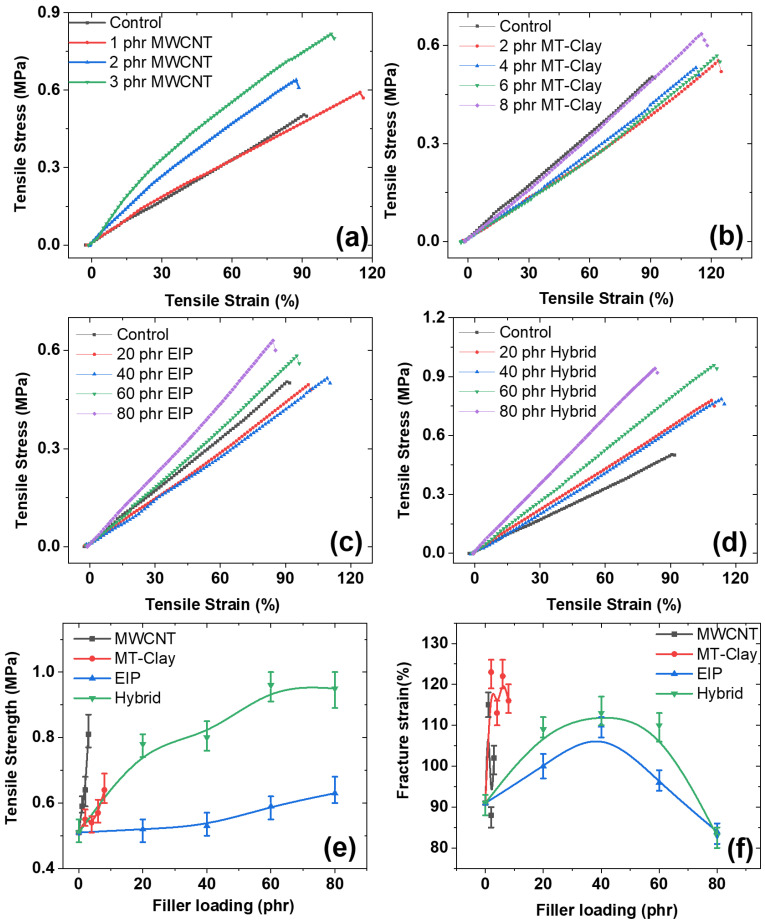
Stress–strain properties of the composites for (**a**) MWCNT; (**b**) MT-Clay; (**c**) EIP; (**d**) Hybrid filler; and (**e**) Tensile strength of different composites; (**f**) Fracture strain of composites.

The effect of filler concentration and tensile strain on the mechanical properties was investigated and is presented in Figure 4a–d. The stress–strain curves reveal that the tensile stress increases with increasing strain until it reaches its maximum at the point of failure. This behavior can be attributed to the re-orientation of filler–filler and filler–polymer microstructures in the direction opposing the tensile strain, leading to an increase in stiffness and, consequently, higher tensile stress. 

The reinforcing ability of the fillers is dependent on their type and concentration in the rubber matrix [49,50]. Three types of fillers—MWCNT, EIP, and MT-Clay—were added to the rubber matrix in a single and hybrid state. All three fillers and their hybrid showed reinforcing abilities, with MWCNT being the most effective and EIP the least effective. Notably, MT-Clay improved the tensile strength moderately but significantly improved the fracture strain. However, due to the large particle size of EIP, a higher concentration is required, which is much higher than that of MWCNT and MT-Clay.

Figure 4e,f demonstrates the impact of filler concentration on tensile strength and fracture strain. MWCNT were found to be the most effective at reinforcing the rubber matrix due to their favorable characteristics, such as their tube-shaped morphology [51] These aspects aid in easy dispersion, a high aspect ratio that aids in forming robust filler–filler interconnections at a lower filler content, and a high surface area that facilitates higher interfacial interactions and leads to better properties [52,53,54]. Additionally, it is worth noting that the hybrid filler exhibits more robust mechanical properties than the three fillers used separately. Moreover, the hybrid filler displays a form of synergism in mechanical properties, with the tensile strength and fracture strain of the filled composites being higher in the hybrid filler than in MWCNT, MT-Clay, and EIP as single fillers. Therefore, it can be concluded that the hybrid filler system should be preferred over the single fillers used in this study.

### 3.5. Theoretical Modeling for Determining the Moduli of the MREs

The present study includes theoretical modeling to validate the experimental results using existing theoretical models. The Guth–Gold Smallwood model [55] and Halpin–Tsai theoretical model [56] are commonly used in literature for theoretical predictions, and their predictions strongly depend on morphological aspects of the filler such as aspect ratio, as well as the volume fraction of the filler [55,56]. The following equation was used for the Guth–Gold Smallwood prediction:

E_1_ = E_o_ [(1 + 0.67 f_1_ϕ_1_)] (1)

E_2_ = E_o_ [(1 + 0.67 f_2_ϕ_2_] (2)

E_3_ = E_o_ [(1 + 0.67 f_3_ϕ_3_] (3)

E_1+2+3_ = E_o_ [(1 + 0.67 f_1_ϕ_1_) + (1 + 0.67 f_2_ϕ_2_) + (1 + 0.67 f_3_ϕ_3_)] × i (4)

E_1_, E_2_, E_3_, and E_1+2+3_ are the predicted theoretical moduli for MWCNT, MT-Clay, EIP, and their hybrid filler system, respectively. E_o_ is the experimental modulus of unfilled rubber. The f_1_, f_2_, and f_3_ are the aspect ratios of the fillers. The ϕ_1_, ϕ_2_, and ϕ_3_ are the volume fractions of the fillers. Moreover, the “i” is the interactive factor among the respective fillers in the hybrid system. 

For the Halpin–Tsai theoretical model, the following equations are used—

E_1_ = E_o_ [(1 + 2 f_1_ϕ_1_)/(1-ϕ_1_) (5)

E_2_ = E_o_ [(1 + 2 f_2_ϕ_2_)/(1-ϕ_2_) (6)

E_3_ = E_o_ [(1 + 2 f_3_ϕ_3_)/(1-ϕ_3_) (7)

E_1+2+3_ = E_o_ [(1 + 2 f_1_ϕ_1_)/(1 − ϕ_1_) + (1 + 2 f_2_ϕ_2_)/(1 − ϕ_2_) + (1 + 2 f_3_ϕ_3_)/(1 − ϕ_3_)] × i (8)

**Figure 5:**
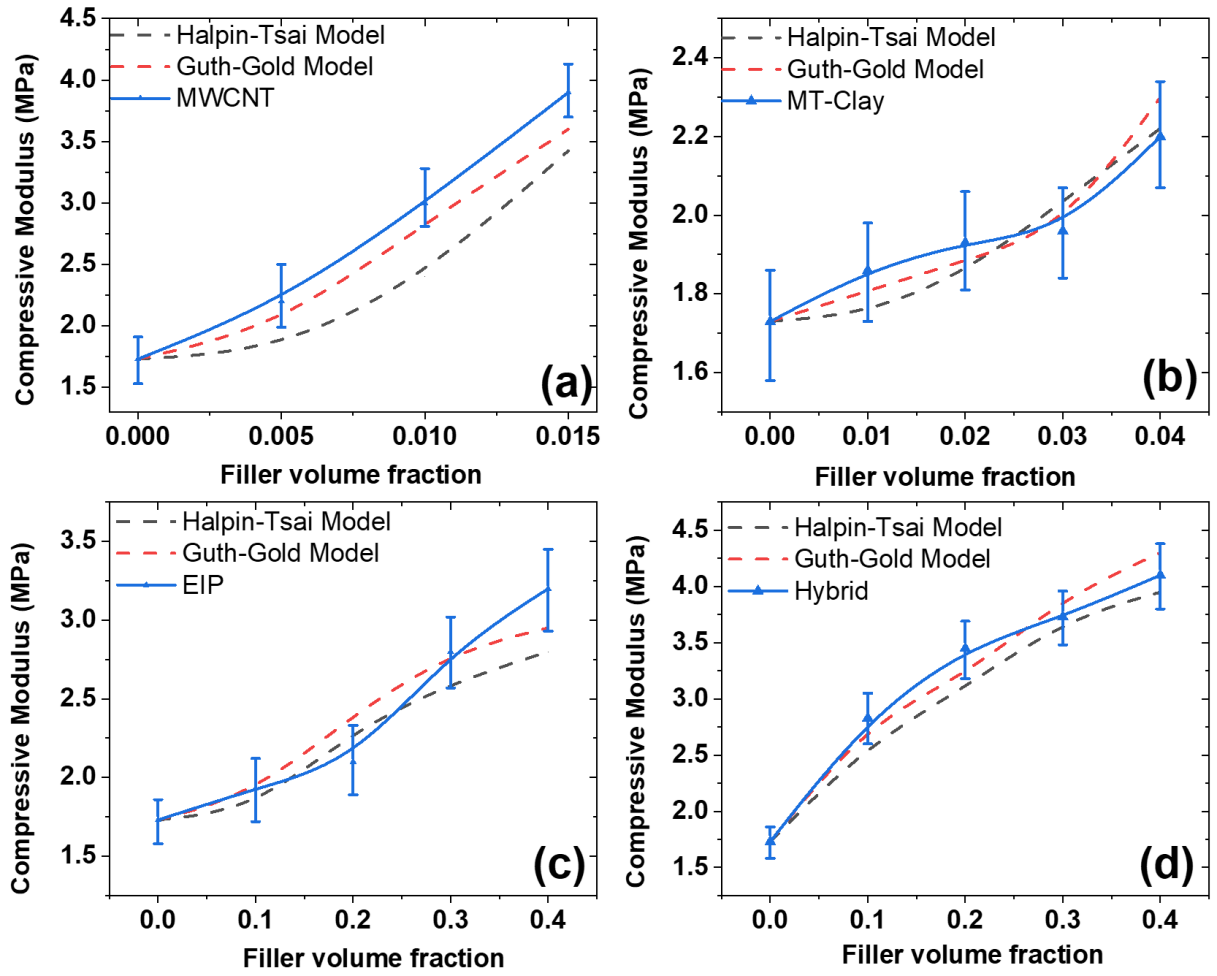
The theoretical and experimental fittings: (**a**) models for MWCNT; (**b**) models for MT-Clay; (**c**) models for EIP; (**d**) models for the hybrid filler system.

In this proposed theoretical model, the components have the same nomenclature as described in the Guth–Gold Smallwood equation. Figure 5a–d indicates that both models agree well with the experimental findings, further validating our results. However, in Figure 5c, the experimental data only agree up to 60 phr of EIP and then deviate. This behavior could be due to differences in assumptions made by the models, such as assuming perfect interfacial bonding between the filler–polymer interface [57] and perfect filler dispersion in the rubber matrix, which is difficult to achieve experimentally. Therefore, there is a deviation between the experimental data and the theoretical models. Additionally, it is worth noting that the hybrid filler system shows synergistic mechanical properties and is therefore more advantageous than using single-filled systems in the composites.

### 3.6. Experimental Deviation from Statistical Average in Hybrid Composites

**Figure 6:**
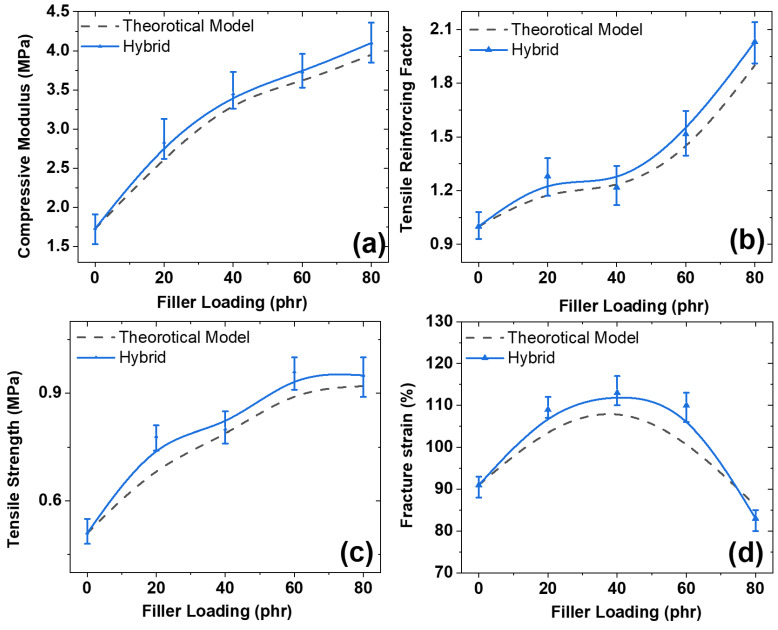
Experimental behavior against theoretical models: (**a**) for compressive modulus; (**b**) for tensile reinforcing factor; (**c**) tensile strength; (**d**) fracture strain.

The experimental behavior and its deviation from the theoretical values have been well-studied in the literature [57,58]. In this work, we use a simple theoretical model based on statistical averages to predict the mechanical properties of the hybrid composites [58]. The compressive behavior shown in Figure 6a can be derived from the following equation:

E_1+2+3_ = [0.1 × E_1_ + 0.4 × E_2_ + 1.5 × E_3_] × i (9)

where “E_1_” is the theoretical modulus of MWCNT, “E_2_” is the theoretical modulus of MT-Clay, and “E_3_” is the theoretical modulus of EIP-based composites. “E_1+2+3_” is the theoretical modulus for hybrid composites containing all three components. In this theoretical model, the constants of 0.1 for 1 phr of MWCNT, 0.4 for 4 phr of MT-Clay, and 1.5 for 15 phr of EIP are related to the filler content in the sample and are used to predict mechanical properties through statistical averages for hybrid composites [58]. The interactive factor “i” considers the dispersion state and filler interactions in the composite. A low value of “i” (i = 0.1) indicates poor filler dispersion and interactions, while a high value of “i” (i ≥ 0.9) indicates good filler dispersion and interactions in the rubber matrix. For the determination of the compressive modulus, the value of “i” was found to be in the range of 0.7 to 0.8. It is worth noting that the literature has extensively studied experimental behavior and its deviation from theoretical values [57,58].

The determination of the tensile reinforcing factor can be derived from the following equation—

R.F._1+2+3_ = [0.1 × R.F._1_ + 0.4 × R.F._2_ + 1.5 × R.F._3_] × i (10)

Here, “R.F._1+2+3_” is the reinforcing factor for hybrid components. “R.F._1_,” “R.F._2_,” and “R.F._3_” are the reinforcing factors of the individual components. Moreover, the interacting factor for determining theoretical R.F. was in the range of 0.5 to 0.8. 

Similarly, the tensile strength in Figure 6c can be derived theoretically from the following equation—

T.S._1+2+3_ = 0.1 × T.S._1_ + 0.4 × T.S._2_ + 1.5 × T.S._3_ × i (11)

where “T.S._1+2+3_” is the theoretical tensile strength of the hybrid system, while “T.S._1_,” “T.S._2_,” and “T.S._3_” are the tensile strength of the individual components. Moreover, the interacting factor for determining the theoretical T.S. was in the range of 0.65 to 0.7.

Finally, the fracture strain in Figure 6d can be derived from the following equation—

F.S._1+2+3_ = 0.1 × F.S._1_ + 0.4 × F.S._2_ + 1.5 × F.S._3_ × i (12)

where “F.S._1+2+3_” is the theoretical fracture strain of the hybrid system, while “F.S._1_,” “F.S._2_,” and “F.S._3_” are the fracture strain of the individual components. Moreover, the interacting factor for determining the theoretical F.S. was around 0.5. From Figure 6a–d, it can be hypothesized that the theoretical models fit well with the experimental findings and are thus useful for further considerations in the literature.

### 3.7. Reinforcing Factor and Reinforcing Efficiency of the Fillers in MREs

**Figure 7:**
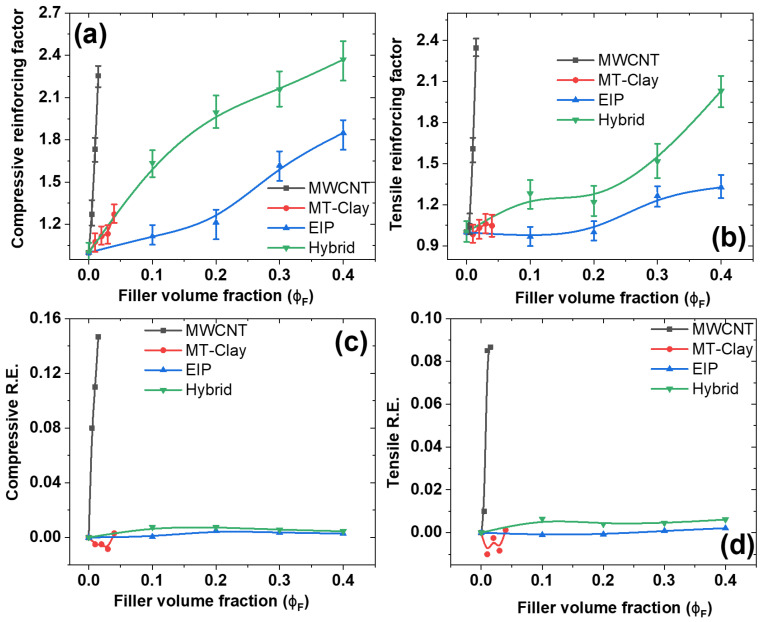
(**a**) R.F. under compressive strain; (**b**) R.F. under tensile strain; (**c**) R.E. under compressive strain; (**d**) R.E. under tensile strain.

Reinforcement via particulate filler in polymer composites is well documented [59]. It is known that fillers with small particle sizes produce higher reinforcement, so studying the reinforcing properties in rubber composites is important for understanding their mechanical properties, such as stiffness, stretchability, tensile strength, and modulus [60,61]. This study analyzed four categories of fillers with different concentrations for their reinforcing effect and efficiency, which is presented in Figure 7. The reinforcing factor of the composites can be calculated using the following equation:

(13) $$R.F.=\frac{EF}{Eo}$$

Here, R.F. is the reinforcing factor, EF is the modulus of the filled composites, and E_o_ is the modulus of the unfilled composites. As shown in Figure 7a,b, the R.F. strongly depends on the type of filler. For example, MWCNT, with its small particle size and higher aspect ratio, was found to be the most promising source of reinforcement in the silicone rubber matrix. Other fillers such as MT-Clay provide medium reinforcement, while EIP, with its large particle size, shows poor reinforcing ability and is thus used in very high amounts compared to MWCNT to obtain optimum reinforcement [61]. Besides R.F., reinforcing efficiency (R.E.) is a significant parameter that affects the mechanical properties of the composites. It is also interesting to note that the R.E. is directly correlated with the concentration of the filler in the composites. The equation for calculating R.E. [62] is

(14) $$R.E.at\;compressive\;strain=\frac{\sigma \left(35\%\right)filled-\sigma \left(35\%\right)unfilled}{wt\%\;of\;filler}$$

(15) $$R.E.at\;tensile\;strain=\frac{\sigma \left(80\%\right)filled-\sigma \left(80\%\right)unfilled}{wt\%\;of\;filler}$$

where “σ” is the stress at a particular strain. The stress values used for calculating R.E. were 35% and 80% for compressive and tensile strain tests, respectively, as obtained from the stress–strain curves in Figure 3 and Figure 4. Notably, MWCNT-based composites exhibited superior R.E. compared to MT-Clay and EIP particles, which can be attributed to their high aspect ratio, tube-shaped morphology, and higher interfacial area with the rubber matrix. These factors allowed for easy dispersion and stronger reinforcement, as seen in Figure 7.

## 4. Applications

### 4.1. Energy Harvesting Applications for the MREs

Energy harvesting using eco-friendly composites is a promising area of study for society. In this study, we fabricated an energy-harvesting device comprising conductive copper electrodes sandwiched with different substrates. The energy generated was due to the dielectric property of the elastomer used in the substrate against mechanical compressive loading, which was kept constant at 30% for all samples [63]. Although piezoelectric materials like PZT [64] or barium titanate [65] have shown promise for high-voltage generation, their use is limited due to their poisonous effects [65,66]. Recently, eco-friendly composite-based energy harvesting has been reported [67].

**Figure 8:**
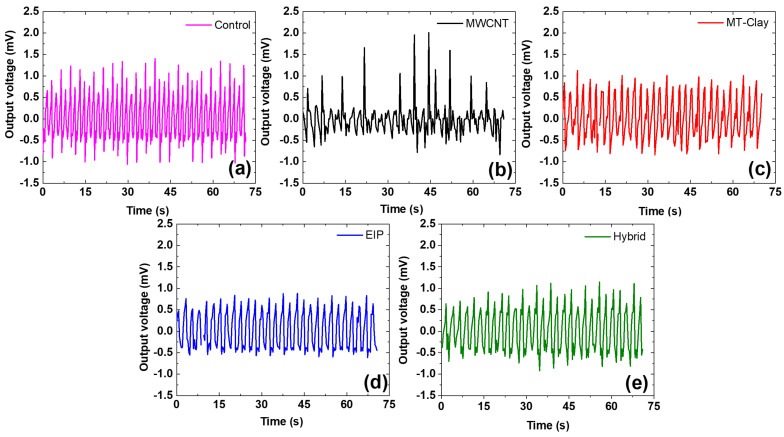
Energy harvesting using different substrates: (**a**) control sample; (**b**) 1 phr of the MWCNT-filled substrate; (**c**) 4 phr of the MT-Clay-filled substrate; (**d**) 40 phr of the EIP-filled substrate; (**e**) 40 phr of the hybrid substrate.

Figure 8 shows the different energy harvesting output voltages for the different substrates. From these measurements, we found that MWCNT-based substrates showed the highest output voltage while EIP-based substrates showed the lowest among all the substrates studied. However, the voltage stability was found to be less efficient in MWCNT-based substrates than in all other materials studied. Therefore, in conclusion, MWCNT-based substrates have higher voltage generation capabilities but the disadvantage of lower voltage stability. In addition to the type of substrate, electrode area is a critical factor affecting the output voltage. For instance, energy harvesting devices with larger electrode surface areas produce higher output voltages than those with smaller ones. We will explore this effect in our future work. 

### 4.2. Magnetic Effect and Stress Relaxation Applications for the MREs

**Figure 9:**
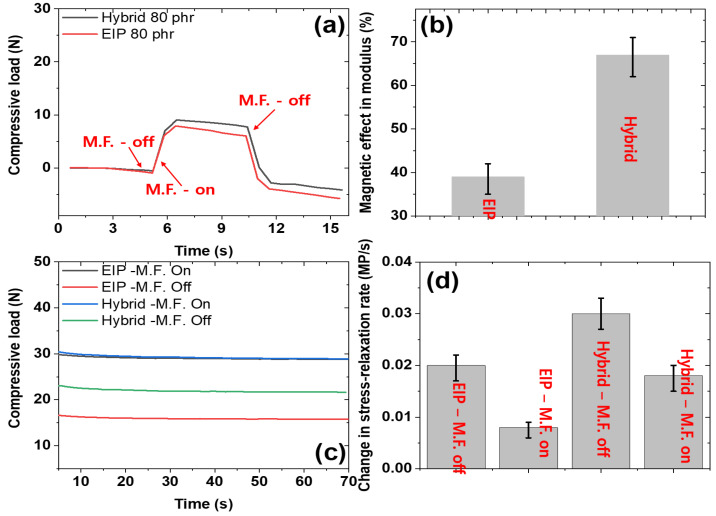
(**a**) Magnetic response of the composites under magnetic switching; (**b**) magnetic effect on moduli of different composites; (**c**) stress relaxation of different composites under magnetic switching; (**d**) change in stress relaxation rate of different composites under magnetic switching.

To magnetic sensitivity measured in this work has been optimized in our previous studies [5]. Figure 9a displays the magneto-mechanical response of the composites during the magnetic switching task. The measurements demonstrate that the compressive load increases when a magnetic field of 100 mT is applied and returns to normal when the magnetic field is turned off. This could be attributed to the orientation of EIP particles in the direction of the applied magnetic field, thereby enhancing the stiffness of the composites [5]. The increase in stiffness is correlated with a rise in compressive load, as shown in Figure 9a. These measurements establish that the composites containing magnetic fillers are sensitive to exposure to a magnetic field, as claimed in the objective of this research. Additionally, it is worth noting that a hybrid-filled composite provides higher sensitivity than using EIP as the only filler. Figure 9b shows the magnetic effect on the moduli of different composites. The magnetic effect was found to be higher for the hybrid composite than for EIP as the only filler. The higher magnetic sensitivity for the hybrid-filled composite could be attributed to the synergistic effect [5] between the MWCNT–EIP fillers, leading to greater sensitivity. Rubber composites reinforced with fillers often exhibit viscoelastic properties that affect their stress relaxation behavior [68]. The effect of magnetic switching and the type of mechanical reinforcement on stress relaxation in rubber composites is shown in Figure 9c. The results indicate that stress relaxation is higher when the magnetic field is on and is higher for hybrid composites than EIP-only-filled rubber composites. Additionally, the stress relaxation rate, as shown in Figure 9d, is influenced by the type of filler and magnetic switching. The stress relaxation rate is higher when the magnetic field is off and lower when it is on for both EIP-only-filled and hybrid-filled composites. The poor reinforcing and magnetic effect of EIP even at 60 phr filler content leads to a small change in magnetic effect and stress relaxation in composites. Moreover, these experiments were performed multiple times to make sure that the conclusions are convincing. Furthermore, hybrid-filled composites exhibit higher stress relaxation rates than EIP-only-filled composites. These results are consistent with the magnetic sensitivity tests shown in Figure 9a,b. The addition of reinforcing fillers, such as MWCNT, improves damping properties in MREs [69]. Therefore, the hybrid filler is the best candidate for achieving improved magnetic sensitivity and good damping in MREs.

## 5. Conclusions

This study demonstrates that incorporating a triple-filler system into silicone rubber has the potential to yield superior mechanical performance, magnetic sensitivity, and energy generation. To this end, composite materials were prepared via solution mixing of MWCNT, MT-Clay, and EIP fillers in both single and hybrid states into the silicone rubber matrix. The improved mechanical performance of the resulting composites was then investigated and reported in this study. Specifically, mechanical stretchability was measured and found to be 91% (control), 102% (MWCNT composites, 3 phr), 116% (MT-Clay composites, 8 phr), 110% (EIP composites, 40 phr), and 113% (hybrid composites, 40 phr). The tensile strength was also analyzed and found to be 0.51 MPa (control), 0.81 MPa (MWCNT composites, 3 phr), 0.64 MPa (MT-Clay composites, 8 phr), 0.63 MPa (EIP composites, 80 phr), and 0.95 MPa (hybrid composites, 80 phr). Furthermore, the effect of the mechanical properties on magnetic sensitivity was explored, and it was found that EIP composites exhibited higher magnetic sensitivity than hybrid composites. However, the latter was identified as the most promising filler system due to its good reinforcement, optimum stiffness, and reasonable magnetic sensitivity. The key takeaway from this study is that selecting a hybrid filler system can result in balanced overall properties that are useful for different applications such as magnetic sensitivity or energy harvesting. For example, the triple-filler system was found to offer good reinforcement from MWCNT, stretchability from MT-Clay, and magnetic sensitivity from EIP in the composite material.

## Figures and Tables

**Table 1:** Fabrication of the different rubber composites.

Samples	RTV-SR (phr)	MWCNT (phr)	MT-Clay (phr)	EIP (phr)	Vulcanizing Solution (phr)
Control	100	-	-	-	2
RTV-SR/MWCNT	100	1, 2, 3	-	-	2
RTV-SR/MT-Clay	100		2, 4, 6, 8		2
RTV-SR/EIP	100			40, 60, 80, 100	2
RTV-SR/Hybrid *	100	1	4	35, 55, 75, 95	2

* The formulation of the filler loadings in the hybrid sample was based on their near-to-percolation value, at which the properties are improved significantly and a dominating effect of the filler can be observed.

## Data Availability

The data presented in this study are available on request from the corresponding author.
